# Meetings of Societies

**Published:** 1901-03

**Authors:** 


### MEETINGS OF SOCIETIES.

### Bristol Medico-Chirurgical Society.

*December 14th, 1900.*

Dr. D. S. Davies, President, in the Chair.

The President proposed that a vote of thanks be given to Mr. J. Paul Bush for his very interesting exhibition of lantern slides, illustrating scenes during the Boer war, which he had given to the Society at the last meeting.—Dr. B. M. H. Rogers seconded the resolution, which was cordially passed.

Mr. A. W. Prichard showed a curious case of hypertrophy of the hand; a skiagram, a cast, and photographs of the hand were also shown. The man was a stonemason, who had worked chiefly in railway tunnels. He had been well until five and a half years ago, when a heavy crowbar lacerated the ball of his thumb and his forearm. No bones had been broken. Cramp-like pains affected the parts. The thumb and some of the fingers had gradually enlarged, and the former became deformed. He could hold tools until three weeks previously. The skin was glossy over the thumb and fingers. Touch was impaired, and one spot on the thumb was anæsthetic. Pain shot up the inner side of the arm, and there was involuntary twitching of the index finger. A fusiform swelling had formed on the inner side of the arm above the internal condyle. It did not fluctuate, and was the size of a Tangerine orange. There were no enlarged glands. Neuritis appeared to have extended up the side of the neck to the head. The patient was willing to submit to any reasonable measures for relief, and the speaker would be glad to consider any suggestions.—Dr. Michell Clarke thought it possible that the affection was analogous to "elephantiasis neuromatosa," and referred to the illustrations and account of that affection given in the recent work of Alexis Thomson.—Mr. Roger Williams said the enlargement in this case was chiefly of the bones, that of the softer parts seemed secondary. In hypertrophy all parts enlarged symmetrically. There was no bead-like condition of the nerves like that of Von Recklinghausen's disease. After crushing and other injuries, chondromatous growths sometimes developed. The swelling at the elbow seemed secondary to the hand condition, not a direct result of the injury, and he thought it might be a chondro-fibro-sarcoma.

Dr. Theodore Fisher showed a patient with a patent ductus arteriosus. He remarked that his patient showed a sign which Dr. Goodhart, ten years ago, had pointed out to be suggestive of patent ductus arteriosus; viz., a harsh localised murmur continuous in systole and diastole. A case had lately been recorded in the *Edinburgh Hospital Reports* in which by this sign a case had been successfully diagnosed during life. The murmur was continuous rushing sound, heard almost from just after the first sound to the first sound again. A thrill was palpable in the third left interspace. In two cases the thrill had been in the second space, and it was in that space in the case shown and was an inch outside the sternal edge. This murmur was not unlike a venous hum, undulating with the second sound. He had made a note of these characters of the murmur before reading the records mentioned. There was another murmur to be heard in his case, due to congenital or acquired causes. No definite symptoms were complained of, but the patient was not able to run about, and had occasional palpitation. She was a woman of twenty-six years of age. She had had rheumatism seven or eight years previously. The heart was enlarged. It was suggested that enlargement of the right heart helped diagnosis, and he thought it was just as likely to occur in these cases as in those of pulmonary stenosis. Absence of cyanosis had been described in other cases.—Dr. MICHELL CLARKE remarked that the history of rheumatism complicated the case. Continuous murmurs were very unusual from congenital defects. They were to be heard in varicose aneurysms. It was not certain that blood passed into the pulmonary artery from the aorta. He had himself only ventured to diagnose pulmonary stenosis and communication between the cavities of the heart in congenital cases. Many cases of congenital heart disease lived to a good age.—Dr. STACK wondered if the continuous murmur might not be a venous hum, and mentioned two cases in which he had heard loud humming murmurs over the heart, but without thrill, and which he had regarded as venous hums and had not been connected with heart lesions.—Dr. FISHER replied that in his case the murmur was harsher and higher pitched than a venous hum, and was associated with a thrill.

Dr. MICHELL CLARKE showed a case of idiopathic muscular atrophy.

Dr. SYMES showed a specimen of blood from a case of lymphatic leukæmia. The blood count showed that the red cells numbered 789,600 per c.m., and the white cells 176,400. Hæmoglobin, about 30 per cent. The total of white cells was made up as follows:—

| | |
|---|---|
| Large hyaline lymphocytes ... ... ... ... ... | 79 per cent. |
| Small hyaline lymphocytes .. ... ... ... ... | 10.8 ,, |
| Myelocytes ... .. ... ... ... ... ... ... ... | 6.4 ,, |
| Finely granular oxyphil leucocytes ... ... ... | 3 ,, |
| Coarsely granular oxyphils (eosinophils) ... | .4 ,, |
| Eosinophil myelocytes ... ... ... ... ... ... | .2 ,, |

There were a few nucleated red corpuscles. Clinically, the case was characterised by the absence of enlargement of the spleen and lymphatic glands, by extreme anæmia and breathlessness, and by hemorrhages into the skin. It terminated fatally in the third month. —Dr. HARRISON said the patient had shown extreme pallor. The heart sounds had been feeble and the heart dilated. He would have been inclined to diagnose fatty degeneration of the heart. The patient's health had been fair until three months before, but for twelve months had not been robust. She left the hospital at her own request in almost a syncopal condition, and died a day or two later.—Mr. ROGER WILLIAMS remarked that the case showed the value of histological examination of the blood.

Mr. W. ROGER WILLIAMS read a paper on "Medical Science in the Nineteenth Century." He enlarged upon the far-reaching effects upon the advancement of medical science which the promulgation of the cell theory, the germ theory, vaccination, and anæsthesia had produced. Advances had been more due to general evolution than to the transcendent genius of individuals. In 1830, with the manufacture of a serviceable microscope, histology had been born. The philosophical comprehensiveness of Virchow was noticed, and his career outlined. The services of Koch and the inauguration of the germ theory were considered, and the advances which had resulted described.

Dr. J. LACY FIRTH read notes on a case of bilocular hydrocele. The hydrocele was of the intra-pelvic and scrotal variety. The abdominal part of the sac extended nearly to the umbilicus, and occupied the left iliac fossa. There was free fluctuation between the intra- and extra-abdominal portions of the hydrocele. Macewen's view, that these hydroceles resulted from the persistence and distension with fluid of a portion of the funicular process lying along the intra-abdominal portion of the vas deferens, was described. The extra-abdominal sac had been easily and successfully removed by working through the inguinal canal after exposing the external abdominal ring, as in the operation for the radical cure of hernia. The rarity of the affection was alluded to.—Messrs. MUNRO SMITH and PAUL BUSH commented upon the case.

---

*January 9th, 1901.*

Dr. D. S. DAVIES, President, in the Chair.

Dr. PATRICK MANSON, C.M.G., F.R.S., gave a demonstration of the life history of the malarial parasite, illustrated by numerous microscopical preparations and lantern slides.

The PRESIDENT proposed a vote of thanks to Dr. Patrick Manson for the deeply interesting, scientific, and graphic address he had given. —Mr. F. RICHARDSON CROSS seconded the resolution, which was enthusiastically carried.

Dr. ELLIOTT alluded to Dr. Manson's statement that quinine was a specific and certain remedy, and asked why, if that were the case, patients who had had malaria abroad, and been apparently cured by efficient treatment with quinine, were yet liable to have a return of the fever, though they had remained in this country without having a chance of being reinfected.—Dr. WALDO asked for information in regard to the production of hæmoglobinuria in some cases by the use of quinine in the treatment of malaria.—Dr. C. K. RUDGE asked for information as to the origin of the malarial parasite, and as to its natural state.—Dr. G. PARKER inquired why malarial fever had disappeared to so great an extent in England. He considered that cases still occasionally occurred in England.—Mr. WINDSOR AUBREY asked fcr information upon the question of the proper administration of quinine, in order to secure the best results in the treatment of malaria.—Dr. K. WILLS asked whether the different degrees of severity of malarial attacks depended upon differences in the parasites, or upon differences in the susceptibility of the patients.—Dr. PATRICK MANSON, in replying, stated that quinine in some cases of malaria caused the appearance of hæmoglobinuria. It did not do so in India; and during twenty-three years in China, though he had given pounds of quinine, he had not seen a case. But practitioners would not pass a month in West Africa without meeting with cases. It seemed to depend upon unexplained differences in the type of the disease. Quinine cured malaria, and checked the evolution of the parasite, but it had no effect upon the phases of the parasite passed in the mosquito, nor upon latent phases in the human body. We had no knowledge as to the nature of this latent state of malaria. Drainage of the country had an important bearing upon the occurrence of malaria. Anopheles were not uncommon in England. People did not now live in valleys. They did not now suffer more than twenty-four hours from malarial fever without taking quinine, so that the chances of survival of the parasite were very small. A dose of grs. vij. to grs. xv. of quinine should be given two hours before the threatened attack. Give grs. xx. to grs. xxx. in the twenty-four hours for three or four days or a week. Then give once a week, say on Sundays, for three or four months. In serious cases, the sooner you gave it the better. One must see that the patient swallowed the medicine, and that it became absorbed. It should be given in solution so as to ensure absorption. If there was vomiting give it hypodermically, or rather intra-muscularly, and do that boldly. Deep injections were absorbed most effectually. With regard to the question whether malaria was always due to the mosquito, he believed that was the case. The mosquito came out at night, and concealed itself by day. It did its business quietly, without humming. Some people did not feel irritation from the bites, and were apt to think they had not been bitten when they really had been. He compared the susceptibility of patients to their susceptibility, for instance, to opium. The severity of an attack depended upon individual susceptibility and the dose of the poison.

---

*February 13th, 1901.*

Dr. D. S. Davies, President, in the Chair.

Dr. Shingleton Smith proposed that Dr. Beddoe, who had been an ordinary member of the Society from its foundation, should be elected an honorary member. Dr. Waldo seconded the proposition, which will be submitted to the Society in due course.

Dr. W. H. C. Newnham showed two specimens of uterine fibroids which he had removed by intra-peritoneal hysterectomy. He laid emphasis on the fact that the cases were exceptional in that they demanded operation for the relief of serious symptoms. The first case was that of a single woman of thirty-nine years of age, who was a cook, and was dependent upon her earnings for a livelihood. She had had excessive hemorrhage for five years. At the operation the ovaries were not removed. The wound, being a large one, was sutured in three layers. Twenty-one days after the operation the patient was up and about. The second case was that of a woman of thirty-five years of age. She had been married sixteen years, and was childless. Six years ago excessive hemorrhage began. It lasted nine or ten days. The tumour had nearly doubled in size in the last twelve months, and the patient had not been free from hemorrhage for more than three days at a time. The same operation was performed as in the last case. Some difficulty was experienced in separating the bladder. The patient was now convalescent.—Dr. Aust Lawrence agreed with the opinions which had been expressed as to the advisability of interfering surgically in cases of fibroids of the uterus. Gynæcologists were a little suspicious that surgeons too readily resorted to operations in cases of this disease. Gynæcologists were more likely to look at both sides of the question when cases were sent to them in consultation. Only a small number of the cases required operation. The modern operation of intra-peritoneal hysterectomy was as great an improvement on old methods as the modern operation of ovariotomy was as compared with the old operation in which the clamp was used.—Mr. Munro Smith took exception to the last speaker's remarks upon the attitude of surgeons with regard to these cases, and thought gynæcologists were just as likely to operate as surgeons when cases were sent to them with that object in view.—Mr. Roger Williams pointed out that the mortality of hysterectomy had been reduced from 30 per cent.

to 10 per cent. by modern methods of operating. He believed that child-bearing was the great prophylactic of fibroid disease. The disease seemed to be increasing in frequency, and this was probably the result of late marriages.—Dr. W. C. SWAYNE stated that in his experience about half the cases were in patients who had borne children, and pointed out that pregnancy was much less likely to occur if fibroids were present in the uterus. Such tumours might be present, though one was unable to detect them. He praised the three-plane method of suturing the parietes, regarding it as less likely to lead to adhesions of the intestine to the scar.—Mr. PAUL BUSH objected to Dr. Lawrence's view that gynæcologists were better qualified than surgeons to treat cases of this disease judiciously.—Dr. K. WILLS described the vaginal operation he had seen adopted for these cases by Prof. Wertheimer, of Vienna. In one case, the tumour removed by that method had been half as large again as Dr. Newnham's specimen. No serious bruising of the parts seemed to result from the procedure.—Dr. NEWNHAM, in replying, said he thought fibroids were as frequent in nulliparous as in multiparous women.

Dr. F. H. EDGEWORTH read a paper on a case of purpura hemorrhagica. The patient was a girl, aged 4. She died ten days after admission to the Bristol Royal Infirmary, her illness having begun five weeks before admission. The first trouble had been the formation of swellings in the parotid regions. On admission there were marked pallor, subcutaneous extravasations of blood—many of small extent, some large—enlargement of the spleen, and slight enlargement of the lymphatic glands in the neck. There was a trace of albumin in the urine. The gums were not spongy. The temperature ranged from 99° to 101°. The blood showed considerable leucocytosis as well as anæmic changes. A week later there was some hemorrhage from the rectum, nose, and gums. At the *post-mortem* examination there were found subserous hemorrhages in the pleuræ, lungs, and dura mater, and large and numerous hemorrhages in the kidneys. Streptococci and staphylococci were found in the blood of the spleen and heart. It was interesting to note that the above symptoms began with enlargement of the parotid glands. Bacteriologically, the case had been one of septicæmia. The existence of leucocytosis accorded with this. The anæmia was chiefly due to the hemorrhage, as shown by the fact that the hæmoglobin-richness, and the number of blood corpuscles were each reduced 30 per cent. The endothelial lining of the capillaries had been damaged by the circulation of septic poison in the blood stream. The organisms mentioned, one or the other, or both, were usually found in the blood in these cases. The non-suppurative parotitis had preceded the other symptoms by some weeks. It was probably septic in origin. Septic parotitis sometimes followed abdominal operations. No wound had been found by which the poison might have been introduced into the system. In diagnosis, malignant endocarditis, and certain specific fevers, especially typhoid, and in this case also, in the early stage, mumps, had to be differentiated from purpura hemorrhagica. The majority of cases in which purpura was associated with hemorrhage from mucous membranes were septicæmic in origin. The prognosis was worse where there were other manifestations of the disease as well as hemorrhages—*e.g.*, enlargement of the spleen. If malignant endocarditis was present the issue would be fatal.—Dr. THEODORE FISHER suggested that the renal changes present might possibly have assisted in causing the hemorrhages, and alluded to the different forms of renal disease and their bearing in the production of hemorrhages.—Dr. ELLIOTT remarked that purpura hemorrhagica must be regarded as a specific fever of some kind. He considered that the prognosis was favourable on the whole, if endocarditis was absent. Nasal bleeding might lead to hæmatemesis. The best treatment was good feeding and tonics.—Dr. W. C. SWAYNE alluded to a case he had once seen at the Evelina Hospital, in which there had been hemorrhage into the subcutaneous tissues and from mucous membranes. The boy died in forty-eight hours. The case had been regarded as one of acute scurvy, but he thought that it was more likely to have been one of septic purpura. He also mentioned a case of puerperal fever he had seen in which the spleen was enlarged, and in which Prof. Kent had found numerous streptococci in the uterine discharge.—Dr. EDGEWORTH, in replying, said he had not gone into the question of treatment, as it was that of septicæmia. The hemorrhage did not often cause death directly. If very persistent the use of turpentine internally was recommended, as advocated by Dr. Gee.

Dr. H. WALDO read notes on a case of hæmatoporphyrinuria. The patient was a man of thirty-three. He had taken drugs for insomnia, and latterly had been very partial to sulphonal. He had consulted Dr. Waldo for giddiness, nervousness, and nausea. Later, severer vomiting and pain from gastritis came on, lasting a week. For a day or two he had been fed on nutrient enemata. Nervous symptoms with fleeting delirium, not unlike *delirium tremens*, followed. Then he became violent. Then the urine became like port wine in colour, with absence of albumin and of red blood-cells. Later, severe epileptic convulsions came on and lasted a week. The patient became weaker and weaker. The pulse rate gradually increased from 80 to 160, and the heart sounds became hardly audible. Nasal feeding had to be resorted to, as food given by the mouth passed into the larynx. The fundus of the eye was normal. The patient died comatose. Probably sulphonal was the cause of the symptoms. Hæmatoporphyrinuria was sometimes met with in health, and in rheumatism, pneumonia, and peritonitis. A case had been published in which convulsions and death resulted from a patient taking grs. xxx of sulphonal in two doses. Dr. Young and Mr. Stoddart relied chiefly on the spectrum produced by the substance for its detection.—Prof. KENT remarked that hæmatoporphyrin was free from iron. The appearance of the spectrum differed in acid and alkaline solutions. Though urine had an acid reaction, the spectrum yielded, when hæmatoporphyrin was present, was that spectrum characteristic of the substance when in an alkaline medium. It had been so in Dr. Waldo's specimen.

The President, Dr. D. S. DAVIES, made some remarks upon plague, and invited Prof. Stanley Kent to show microscopic specimens and cultures of the bacillus pestis. The question of plague was getting more and more important. Since the Hong-Kong epidemic in 1896 the disease had assumed a pandemic character. Since the seventeenth century the area affected had gradually retreated eastwards. Plague had disappeared from the east of Europe in 1840. This retreat eastwards was probably due to the fact that the distribution of this disease depended upon the distribution of a sub-family of rats. Probably, when the bacillus became less virulent, it could only survive in animals which were specially prone to receive it. In Glasgow the rats had escaped infection. Plague in the human subject should be easily controlled, like typhus and small-pox. If rats became infected control was more difficult, and danger arose from the public being indifferent to the danger of the importation of plague by animals. Cholera, on the other hand, was a distinctly human disease, and for protection against it no special regulations affecting animals were required. Experience showed that in the dissemination of plague rats were dangerous. To prevent rats passing to the land, ships should be moored two feet away from the quay, rat-guards should be fixed on the cables passing to the quay, and special watch should be kept to prevent the migration. It would be useful if there were powers to disinfect ships after the discharge of their cargo. Mention was then made of a steamboat which had brought a cargo of maize to Bristol from the River Plate, an infected port. Suspicion was aroused by finding twelve dead rats in one of the holds of the vessel. One of these rats had been examined by Prof. Kent, who reported that the appearances did not exclude plague. Two of the rats were then sent to Prof. Klein, who concluded that the plague virus was present in them. Prof. Klein had also found the bacillus in a Cardiff rat.—Prof. KENT pointed out that plague might be diagnosed by ordinary symptoms or by bacteriological investigations. The best material to obtain for the latter purpose was fluid from a bubo. If it was difficult to get any fluid, a little salt solution might be first injected. If there were no bubo, the blood might be examined. A blood film did not usually show the organism unless the cases were of very virulent type. Sputum was not so good for these tests. The organisms existed in enormous numbers in the dejecta. The *bacillus pestis* was a very small organism, consisting of short rods with rounded ends. It was so short that it might appear like a coccus. A staining feature—though not quite a constant one—was that the middle of the rods stained less than the ends (bipolar staining). The appearances of cultures in various media were then described. Almost all animals were susceptible. When affected they became dull, and they refused food and their hair stood on end. They usually died in convulsions in three to five days. Prof. Kent then showed cultures and microscopical preparations, illustrating the subject. Some of these preparations had been obtained from the dead rat found in the ship in Bristol, as described above.

J. LACY FIRTH.

